# Gene module regulation in dilated cardiomyopathy and the role of Na/K-ATPase

**DOI:** 10.1371/journal.pone.0272117

**Published:** 2022-07-28

**Authors:** Yingnyu Gao, Lilian N. D. Silva, John D. Hurley, Xiaoming Fan, Sandrine V. Pierre, Komal Sodhi, Jiang Liu, Joseph I. Shapiro, Jiang Tian

**Affiliations:** 1 Marshall Institute for Interdisciplinary Research, Marshall University, Huntington, WV, United States of America; 2 Department of Biomedical Sciences, Marshall University Joan C. Edwards Medical School, Huntington, WV, United States of America; 3 Department of Medicine, University of Toledo, Toledo, OH, United States of America; King’s College London, UNITED KINGDOM

## Abstract

Dilated cardiomyopathy (DCM) is a major cause of cardiac death and heart transplantation. It has been known that black people have a higher incidence of heart failure and related diseases compared to white people. To identify the relationship between gene expression and cardiac function in DCM patients, we performed pathway analysis and weighted gene co-expression network analysis (WGCNA) using RNA-sequencing data (GSE141910) from the NCBI Gene Expression Omnibus (GEO) database and identified several gene modules that were significantly associated with the left ventricle ejection fraction (LVEF) and DCM phenotype. Genes included in these modules are enriched in three major categories of signaling pathways: fibrosis-related, small molecule transporting-related, and immune response-related. Through consensus analysis, we found that gene modules associated with LVEF in African Americans are almost identical as in Caucasians, suggesting that the two groups may have more common rather than disparate genetic regulations in the etiology of DCM. In addition to the identified modules, we found that the gene expression level of Na/K-ATPase, an important membrane ion transporter, has a strong correlation with the LVEF. These clinical results are consistent with our previous findings and suggest the clinical significance of Na/K-ATPase regulation in DCM.

## Introduction

Heart failure affects more than 40 million people globally with a high rate of morbidity and mortality [1, 2]. Cardiac hypertrophy is common in the early stage of heart failure progression, whereas dilated cardiomyopathy (DCM) is the leading cause of heart transplantation [3]. Clinical data suggested that about 50% of patients with cardiac hypertrophy eventually become decompensated and develop heart failure, while the other 50% develop diastolic dysfunction [4–6]. Initial cardiac hypertrophy is a mechanism that compensates for reduced cardiac output, but subsequent cardiac myocyte death, reduced contractility, and massive tissue fibrosis compromise cardiac function and lead to heart failure [4, 5, 7, 8]. It is also known that black people have the highest risk of heart failure-related death [9]. The risk for developing DCM in black people is about 3-fold compared to whites, and the death rate is also higher in black patients that cannot be explained by socioeconomic status [10]. The overall hospitalization rate of heart failure has improved, but the disparity between black and white people has not decreased [11]. Population-based gene-sequencing has identified some variations in the black patient cohort that may be associated with racial differences between blacks and non-Hispanic whites [12, 13]. However, it has not been well understood how these variants increase the risk of heart failure in blacks. The weighted gene co-expression network analysis (WGCNA) is a widely used high-throughput data analysis tool to study biological networks using large cohorts of patient data [14, 15]. The R package of WGCNA allows researchers to define gene modules and study the relationships between co-expressed gene modules and clinical phenotypes as well as to compare the gene module changes between different groups of patients.

From clinical data and animal studies, it has been shown that decrease of Na/K-ATPase is an important risk factor for cardiac decompensation and dysfunction [16–23]. Decrease of Na/K-ATPase has also been a significant phenomenon in patients with aging [24–26], diabetes with hypertension [27–29], and neurological disorders [30, 31]. Na/K-ATPase is an important membrane protein enriched in muscle and kidney tissues. A functional Na/K-ATPase is composed of alpha and beta subunits. There are four alpha isoforms (α1, α2, α3, and α4) and at least three beta isoforms (β1, β2, and β3) that are expressed in a tissue-specific pattern. Human hearts express three alpha isoforms (α1, α2, α3) of Na/K-ATPase [32–34], but the specific role of each isoform is not fully understood. Na/K-ATPase has been extensively studied for its ion transporting function since it was discovered in the 1950’s. It was not until the early 2000’s that the signaling function of Na/K-ATPase started to be appreciated [35–38]. More recently, we demonstrated that reduction of α1 isoform of Na/K-ATPase causes cardiac cell apoptosis in response to its ligand treatment in animal models of uremic cardiomyopathy [19, 20]. In the current work, we used WGCNA as a tool and analyzed RNA-sequencing data in DCM patients and compared the DCM-related gene modules and signaling pathways between African Americans and Caucasian Americans.

## Methods

### Access to patient data

The gene expression FPKM (fragments per kilobase of exon per million reads) data of RNA-sequencing were downloaded from public available NCBI GEO datasets (GSE141910). The clinical phenotype data and gene expression count data of this cohort were obtained from an online source (GitHub—mpmorley/MAGNet), which was kindly provided by Dr. Michael Morley from University of Pennsylvania.

### DESeq2 normalization and pathway analysis

The count values of RNA-sequencing data from heart left ventricle tissue were analyzed for gene expression change using the DESeq2 R package [39] with RStudio [40]. The study cohort included patients with dilated cardiomyopathy (DCM), hypertrophic cardiomyopathy (HCM), peripartum cardiomyopathy (PPCM), and donors of heart transplant. Since the number of HCM and PPCM was relatively small and only DCM patients received heart transplant in this cohort, we used data only from the DCM patients (n = 166) and their donors (n = 166) for this analysis. The differentially expressed genes (DEGs) were defined as log_2_FoldChange > = 1 or < = -1, and p value< = 0.01. Gene names of DEGs were then uploaded to the Enrichr website (https://maayanlab.cloud/Enrichr/) for gene ontology (GO) analysis. The KEGG pathways analysis was performed on the WebGestalt website (www.webgestalt.org) by uploading the whole gene set and their log_2_FoldChange values derived from the Deseq2 analysis.

### Weighted gene co-expression network analysis (WGCNA)

The FPKM values of RNA-sequencing data from GSE141910 dataset were used for gene co-expression network analysis using a WGCNA R package [15]. Gene expression data were checked for extremely low expressing genes or missing values using goodSamplesGenes function in the WGCNA package. The network construction, module detection, topological analysis, and visualization were performed following the online WGCNA tutorial (https://horvath.genetics.ucla.edu/html/CoexpressionNetwork/Rpackages/WGCNA/Tutorials/). Specifically, a weighted gene network was created based on the adjacency matrix, which was calculated from the gene co-expression similarity as described in the original publication of WGCNA R package [15]. The one-step automatic network construction and module detection method were followed from the online tutorial. The soft threshold power was set at 10, the minimum gene module size was set at 30, and the merge cut height was set at 0.25. Detected gene modules were assigned to different colors, and gene names from each module were extracted to a spreadsheet for pathway enrichment analysis. To obtain the relationship of each gene module to the clinical phenotypes, the correlation of each module and the phenotype as well as their p values were calculated using the moduleTraitCor and module TraitPvalue functions in the WGCNA R package. The relationship was presented as a heatmap using the value of coefficient. The continuous and categorical variables from the available clinical phenotypes were used for this relationship analysis. In addition, the Na/K-ATPase gene expression data were also used as a phenotype in this analysis.

### Consensus analysis between African Americans and Caucasians

The FPKM data were sub-grouped into African Americans (AA) and Caucasians (CA). Topological characteristics in the two groups were compared using the aforementioned WGCNA package [14] following the step-by-step instructions. The one-step automatic network construction method WGCNA R package was used for network construction, gene module detection, and consensus analysis. A soft threshold power of 10 was applied for the analysis. The minimum gene module size was set at 30, and the merge cut height was set at 0.25. The consensus gene dendrogram, gene module correlation, and preservation were calculated and presented as indicators of similarity between the AA and CA groups.

### Transcriptional Factor (TF) enrichment analysis using ChEA3 online platform

The ChEA3 TF enrichment analysis provides a platform (http://maayanlab.cloud/chea3/) to allow users to input their interested gene list and identify potential TFs that may coordinate the regulation of the gene list [41]. TFs are prioritized based on the overlap between user-inputted gene sets and annotated sets of TF targets stored within the ChEA3 database. To perform the TF enrichment analysis, we extracted the gene list from the magenta gene module and input to the ChEA3 platform. The TFs that regulate Na/K-ATPase gene expression was then compared between healthy donors and patients.

### Network presentation using Cytoscape program

The top module corresponding to each phenotype was identified and the genes included in these modules were uploaded to the Cytoscape program (version 3.8.1) against STRING database as described in our previous publication [42]. A network indicating potential protein-protein interaction was created and presented as a network map. For Na/K-ATPase-related pathway analysis, the log_2_FoldChange data derived from DESeq2 analysis were uploaded to Cytoscape program and searched against Na/K-ATPase/Src wikipathway (Wikipathway WP5051).

### Statistics

The RNA-Sequencing analysis was performed using a statistical method as we previously reported [39, 42]. Gene expression changes were presented as “volcano” plots with the -log_10_ of the p-value for y-axis, while the log_2_FoldChange for x-axis. A p-value <0.01 was used as a threshold to select the differentially expressed genes (DEGs). The DEGs were then used for gene ontology (GO) analysis. For the KEGG pathway analysis, the entire gene dataset was used so that the GSEA algorithm can apply the log_2_FoldChange data to determine whether gene sets were coordinately over or under-expressed [43]. To construct the unsigned network, we applied soft-thresholding power following the WGCNA R package tutorial. A threshold power of 10 was chosen based on the scale independence and mean connectivity analysis. The relationship between a gene module and clinical phenotype was indicated by the coefficient number and p value calculated using functions from the WGCNA R package.

## Results

### Characteristics of patients and donors

The published RNA-sequencing dataset (GEO141910) is a cohort of heart failure patients including patients who received heart transplant and their donors. The RNA sequencing was obtained from 366 samples of human heart left ventricles. Among these samples, 166 patients were diagnosed with dilated cardiomyopathy (DCM) and received heart transplants, while another 166 samples were from non-failing donors. The data also contained RNA-sequencing data of 28 hypertrophic cardiomyopathy (HCM) and 6 peripartum cardiomyopathy (PPCM) patients. In the cohort, 172 were females, and 124 were African Americans (242 Caucasian Americans). Among African Americans, 77 were DCM, 44 were donors, 1 was HCM, and 2 were PPCM. In Caucasians, 89 were DCM, 122 were donors, 27 were HCM, and 4 were PPCM. The basic characteristics of the cohort is summarized in Table 1.

**Table 1:** Basic characteristics of patients and donors in the database GSE141910.

	Donors (166)	Missing value (number)	Patients (200)	Missing value (number)
**Age (years)**	55.9±14	-	51.1±11.3	-
**African/Caucasian Americans**	44/122	-	80/120	-
**Female**	89	-	83	-
**Dilated Cardiomyopathy**	-	-	166	-
**Hypertrophic cardiomyopathy**	-	-	28	-
**Peripartum cardiomyopathy**	-	-	6	-
**Weight (kg)**	84.9±22.2	-	78.1±21.5	-
**Height (cm)**	167.2±16.7	(1)	170.0±16.7	-
**Heart weight (g)**	424.6±115.2	(7)	504.1±141.2	-
**Left ventricle ejection fraction**	0.56±0.12	(72)	0.18±0.10	(8)
**LV mass (g)**	229.8±69.1	(68)	324.0±93.7	(139)
**AFIB**	17	(1)	89	(4)
**VT/VF**	1	(1)	88	(1)
**Diabetes**	3	(2)	41	-
**Hypertension**	102	(1)	84	-

AFIB, atrial fibrillation; VT/VF, ventricular tachycardia/ventricular fibrillation.

### Gene expression and pathway changes in DCM patients receiving heart transplants compared to non-failing donors

**Fig 1:**
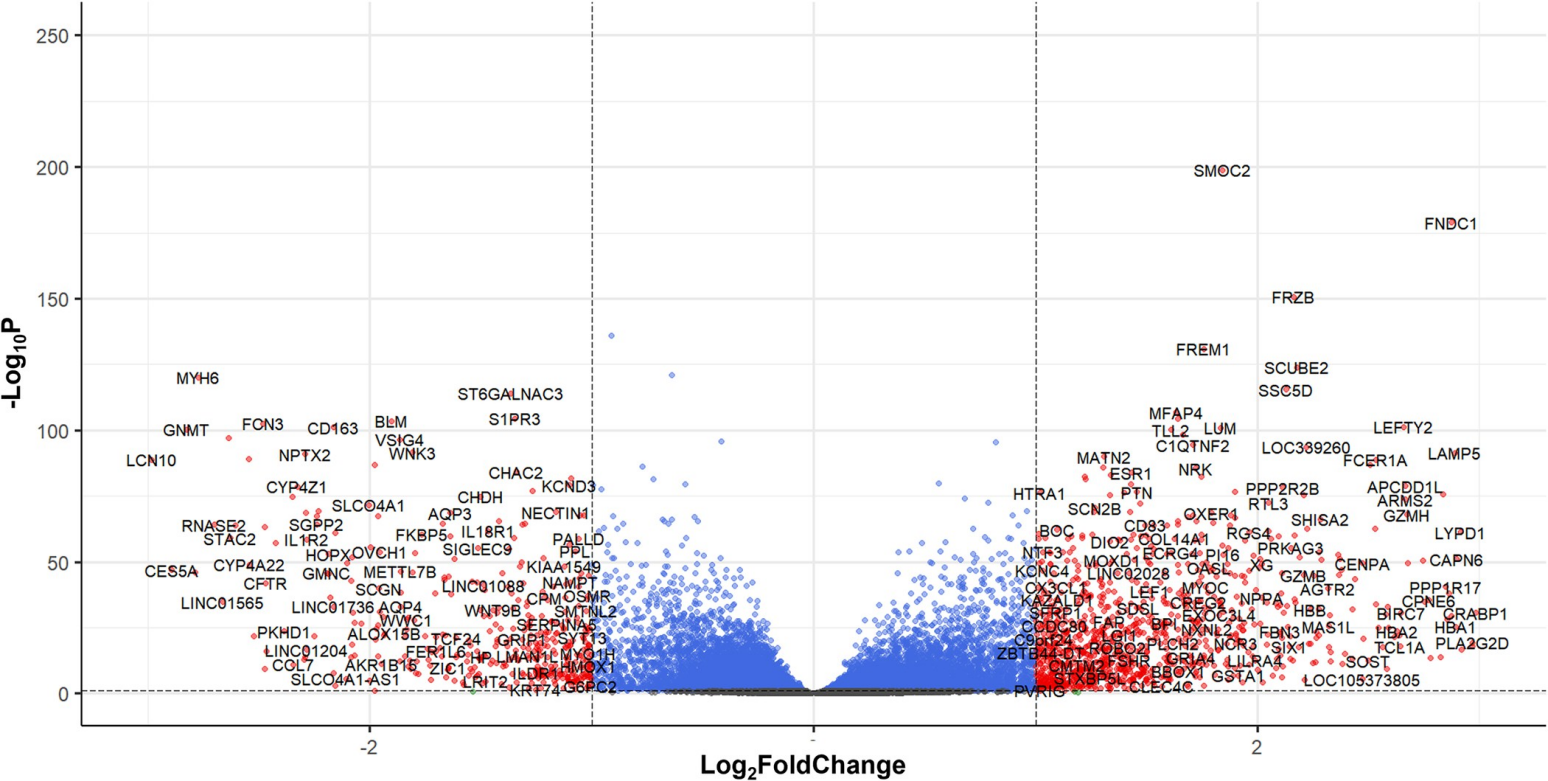
Volcano plot of gene expression changes in DCM patients undergoing heart transplant versus donors. RNA sequencing data from left ventricle heart tissue of GEO dataset GSE141910 was used for foldchange analysis with DESeq2 R package. The volcano plot was created using EnhancedVolcano. The X-axis shows the log_2_FoldChange and the Y-axis shows the -log_10_ of p value. Red color shows genes that are significantly changed (log_2_FoldChange> = 1, or < = -1, and p<0.01). Blue color shows genes that had more than or equal to two-fold change, but p> = 0.01. Black color shows genes that have less than two-fold change and p> = 0.01.

**Fig 2:**
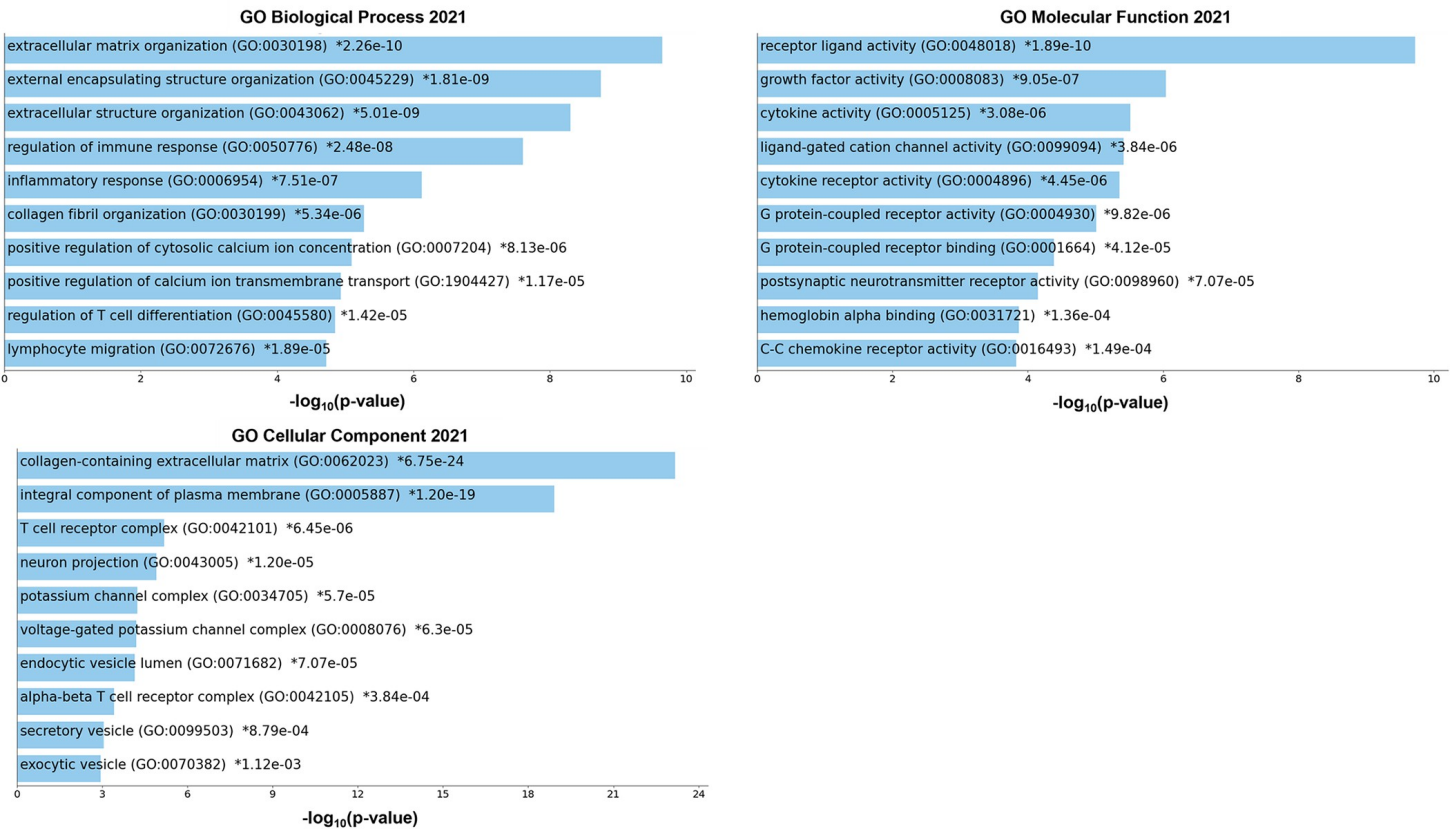
Gene ontology (GO) analysis of differentially expressed genes in DCM patients versus donors. Differentially expressed genes (DEGs) were derived from DESeq2 analysis that had more than or equal to two-fold changes in DCM patients versus donors. The GO analysis was performed using the web based Enrichr program. The figures of overrepresented pathways in GO biological process, molecular function, and cellular component were created by Appyter, an enrichment analysis visualizer.

**Fig 3:**
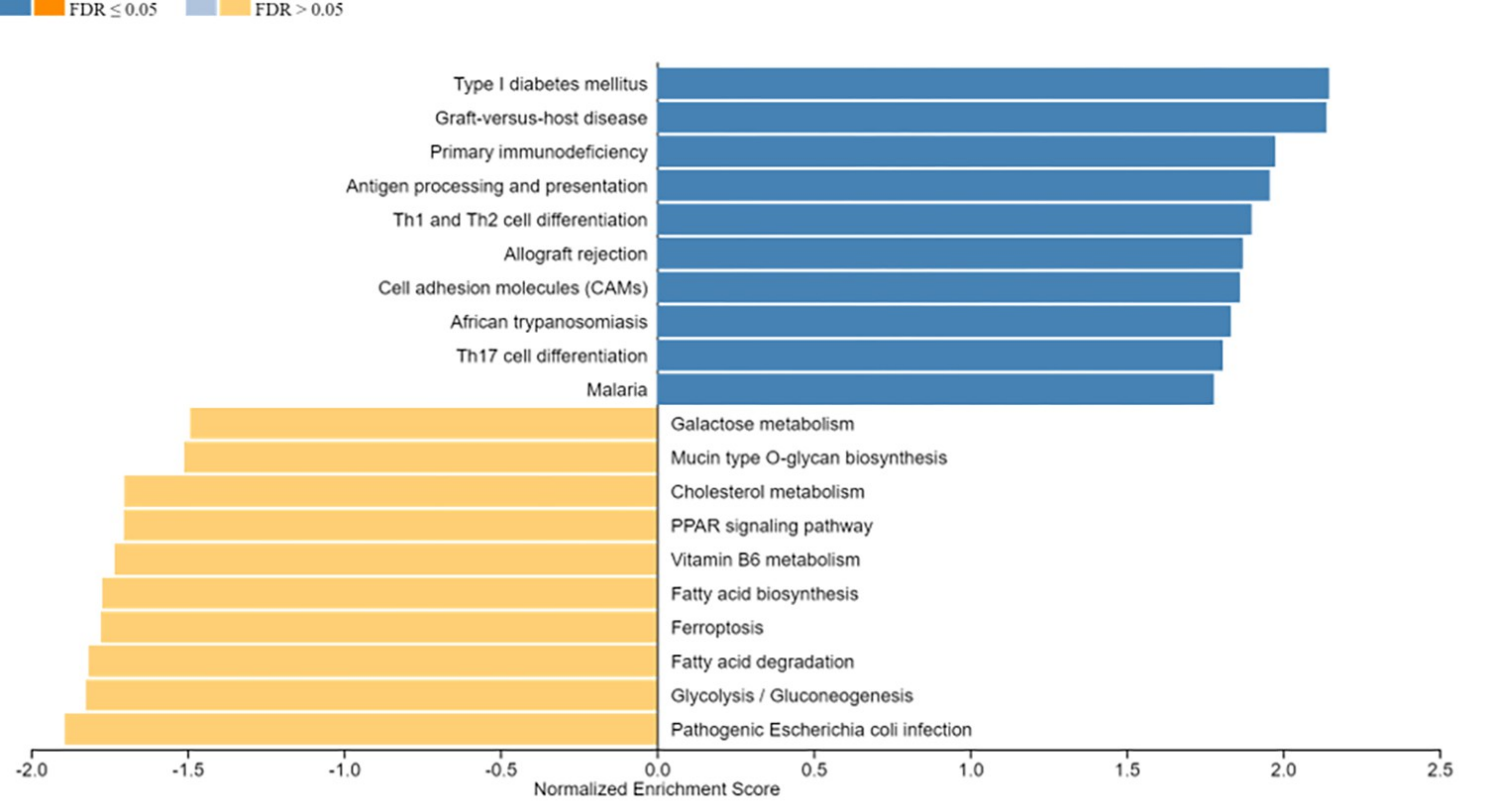
KEGG pathway analysis in DCM patients versus donors. The whole gene set of log_2_FoldChange data from DCM patients versus donors were uploaded to the web based GSEA analysis program (webgestalt.org) for KEGG analysis. The top 10 upregulated (blue) and downregulated (orange) pathways are presented in the figure. FDR, false discovery rate. FDR<0.05 indicates significant change.

We performed the gene expression analysis using DESeq2 based on the published RNA-sequencing data obtained from the GEO141910 cohort. The log_2_FoldChange and statistical significance between DCM patients (n = 166) and their donors (n = 166) derived from the DESeq2 is presented as a volcano plot in Fig 1. The log_2_FoldChange data was used for x-axis and the -log_10_P data was used for y-axis in the volcano plot. To identify the significantly changed genes (DEGs), we applied the threshold as described in the Method section (log_2_FoldChange> = 1 or < = -1 and p value<0.01). A total of 1469 genes were found to be significantly changed in DCM patients versus the donors. These DEGs were then uploaded to the Enrichr webpage for gene ontology (GO) analysis. The top 10 overrepresented pathways based on their p value are shown in Fig 2. Both GO Biological Process and Cellular Component analysis showed that extracellular matrix-related genes were overrepresented in the DCM patients, while the GO Molecular Function showed that different receptor-ligand activity-related and immune activity-related pathways were overrepresented. We also performed a gene set enrichment analysis (GSEA) using the whole set of log_2_FoldChange data derived from DESeq2 analysis. As shown in Fig 3, the pathways of Type I diabetes mellitus, immune system, and cell adhesion molecules related pathways were significantly upregulated while several metabolic pathways were downregulated in DCM patients compared to the donors. However, these downregulated pathways were not statistically significant (FDR>0.5).

### Gene module regulation in dilated cardiomyopathy

**Fig 4:**
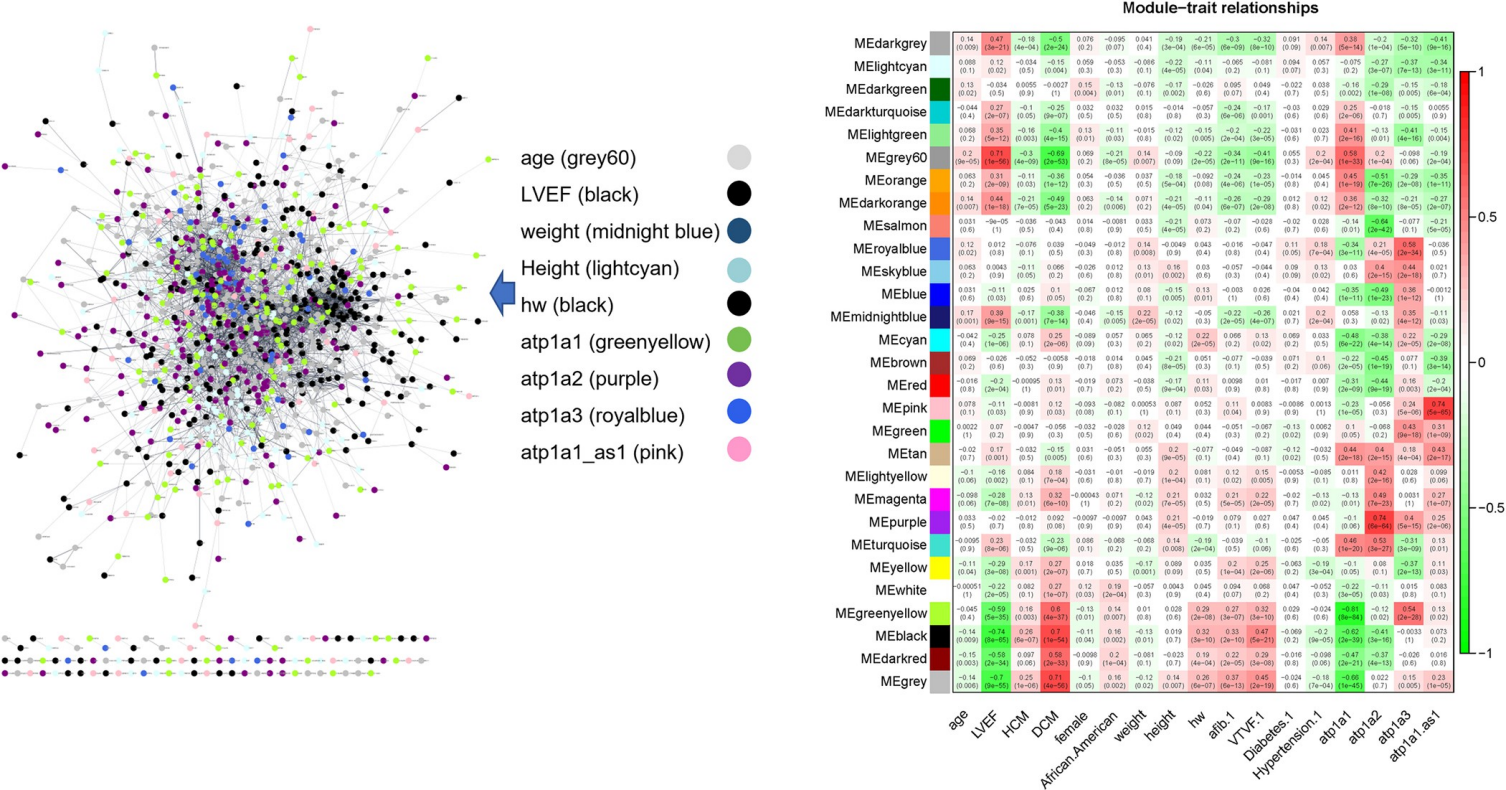
Relationship of gene modules and clinical phenotypes. The weighted gene co-expression network analysis (WGCNA) was performed with the published dataset of GSE141910 using the WGCNA R package. The correlation coefficients of each gene module and corresponding clinical phenotype were calculated and displayed as a heatmap as shown in the right panel of the figure. The green color indicates a negative correlation and red color indicates a positive correlation between modules and phenotypes. The p values are shown in parentheses under each coefficient number. Gene modules were analyzed and presented in different colors as shown on the left side of the heatmap, and the clinical phenotypes were shown at the bottom of the heatmap. The left panel is a String analysis showing the potential protein-protein interactions between the genes that included the top gene modules corresponding to each phenotype. The colors in the String network indicate each clinical phenotype and corresponding gene modules listed in the center of the figure.

To study the co-expressed gene network that may associate with the phenotype of DCM, we performed a weighted gene co-expression network analysis (WGCNA) using the RNA-sequencing data of heart samples from the GEO141910 dataset. We first performed sample clustering and removed 3 outliers from the samples using a cutoff of 220 in the clustering tree. A soft threshold power of 10 and a minimum module size of 30 were applied for gene module detection. The module-trait relationship was analyzed with WGCNA program. We selected only continuous variables in the phenotype data for this analysis. The LV mass data was excluded because it has a significant number of missing values (207 out of 332 were missing). In addition to the clinical phenotypes, we also included the gene expression level of Na/K-ATPase alpha 1, alpha 2, alpha 3 isoforms, and the Na/K-ATPase alpha1 antisense gene (alph1a1-as1) as phenotypes for this relationship analysis. As shown in Fig 4, a total of 29 co-expressed gene modules were detected in this analysis. The correlation coefficients of each gene module and corresponding phenotype were calculated and displayed as a heatmap shown in the right panel of the figure. The blue color indicates a negative correlation, and the red color indicates a positive correlation between modules and phenotypes. The p value is shown in parentheses under each coefficient number. Gene modules were analyzed and presented in different colors as shown on the left side of the heatmap, and the clinical phenotypes are shown at the bottom of the heatmap. Based on this gene module-trait relationship analysis, we found that several gene modules were significantly associated with both the DCM phenotype and left ventricle ejection fraction (LVEF). Since DCM patients had lower LVEF, the correlation coefficient was in the opposite direction for DCM and LVEF. The association to HCM, age, weight, height, and heart weight was generally moderate. Interestingly, we found that several gene modules were also significantly associated with Na/K-ATPase gene expression. Specifically, selected gene modules such as the greenyellow and black modules were significantly related to both LVEF and Na/K-ATPase alpha 1 gene expression. To analyze the relationship of the genes included in these gene modules, we selected the top gene module corresponding to each phenotype and uploaded the genes from these top modules to the Cytoscape program. Using STRING database as a targeted network, we created a protein-protein interaction network map as shown on the left panel of Fig 4. The different colors in the network indicated the genes that included in modules from each phenotype. The network analysis suggested that these genes were highly interactive with each other and may have intrinsic relationship in the regulation of these gene expression.

To further understand how these gene modules are related to the phenotype of DCM, we uploaded the top 5 gene modules (black, greenyellow, darkred, grey60, and grey) with strong association to DCM and LVEF and performed the pathway enrichment analysis using web based Enrichr platform. Gene names from each module were used as input for the pathway enrichment analysis. BioPlanet 2019 pathway database was used for pathway analysis. The top 10 enriched pathways (ranking was based on their p-value) for each gene module are summarized in Table 2. These results showed similar pathways as in GO and KEGG analysis, such as extracellular matrix (ECM)-related and immune response-related pathways, suggesting that fibrosis and inflammation may be common in heart tissue from DCM patients. On the other hand, it revealed that gene module such as the greenyellow module, which contains many small molecule transporters, was also strongly associated with the DCM phenotype and LVEF.

**Table 2:** Top 10 enriched signaling pathways in selected gene modules.

	Black	Greenyellow	Darkred	Grey60	Grey
**1**	Extracellular matrix organization	Proximal tubule bicarbonate reclamation	Interleukin-12-mediated signaling events	TGF-beta regulation of extracellular matrix	Interleukin-5 regulation of apoptosis
**2**	Collagen biosynthesis and modifying enzymes	Physiological and pathological hypertrophy of the heart	Interleukin-2 signaling pathway	Inhibition of matrix metalloproteinases	Interleukin-7 interactions in immune response
**3**	Syndecan 1 pathway	CHL1 interactions	CD8/T cell receptor downstream pathway	Tyrosine metabolism	Cyclin A/B1-associated events during G2/M transition
**4**	TGF-beta regulation of extracellular matrix	Amino acid transport across the plasma membrane	Primary immunodeficiency	FSH regulation of apoptosis	Interleukin-1 regulation of extracellular matrix
**5**	Integrins in angiogenesis	Metapathway biotransformation	T cell receptor signaling in naive CD8+ T cells	Interleukin-4 regulation of apoptosis	Activation of calcium-permeable kainate receptor
**6**	Small leucine-rich proteoglycan (SLRP) molecules	Transmembrane transport of small molecules	T helper cell surface molecules	Pathogenic Escherichia coli infection	ATF2 transcription factor network
**7**	Beta-1 integrin cell surface interactions	Beta-3 integrin cell surface interactions	CTL mediated immune response against target cells	Interleukin-1 regulation of extracellular matrix	G1 to S cell cycle control
**8**	Signaling by PDGF	Growth hormone receptor signaling	T cell receptor signaling pathway	Cytochrome P450 pathway	Potassium channels
**9**	NCAM1 interactions	Platelet amyloid precursor protein pathway	Generation of second messenger molecules	Phase I of biological oxidations: functionalization of compounds	Cyclins and cell cycle regulation
**10**	Keratan sulfate degradation	T cell receptor/Ras pathway	Lck and Fyn tyrosine kinases in initiation of T cell receptor activation	Biological oxidations	Interleukin-4 regulation of apoptosis

CHL1, close homolog of L1; FSH, follicle-stimulating hormone; ATF2, activating transcription factor-2; CTL, cytotoxic T cells; PDGF, platelet-derived growth factor; NCAM1, neural cell adhesion molecule 1.

### Comparison between African Americans and Caucasian Americans

**Fig 5:**
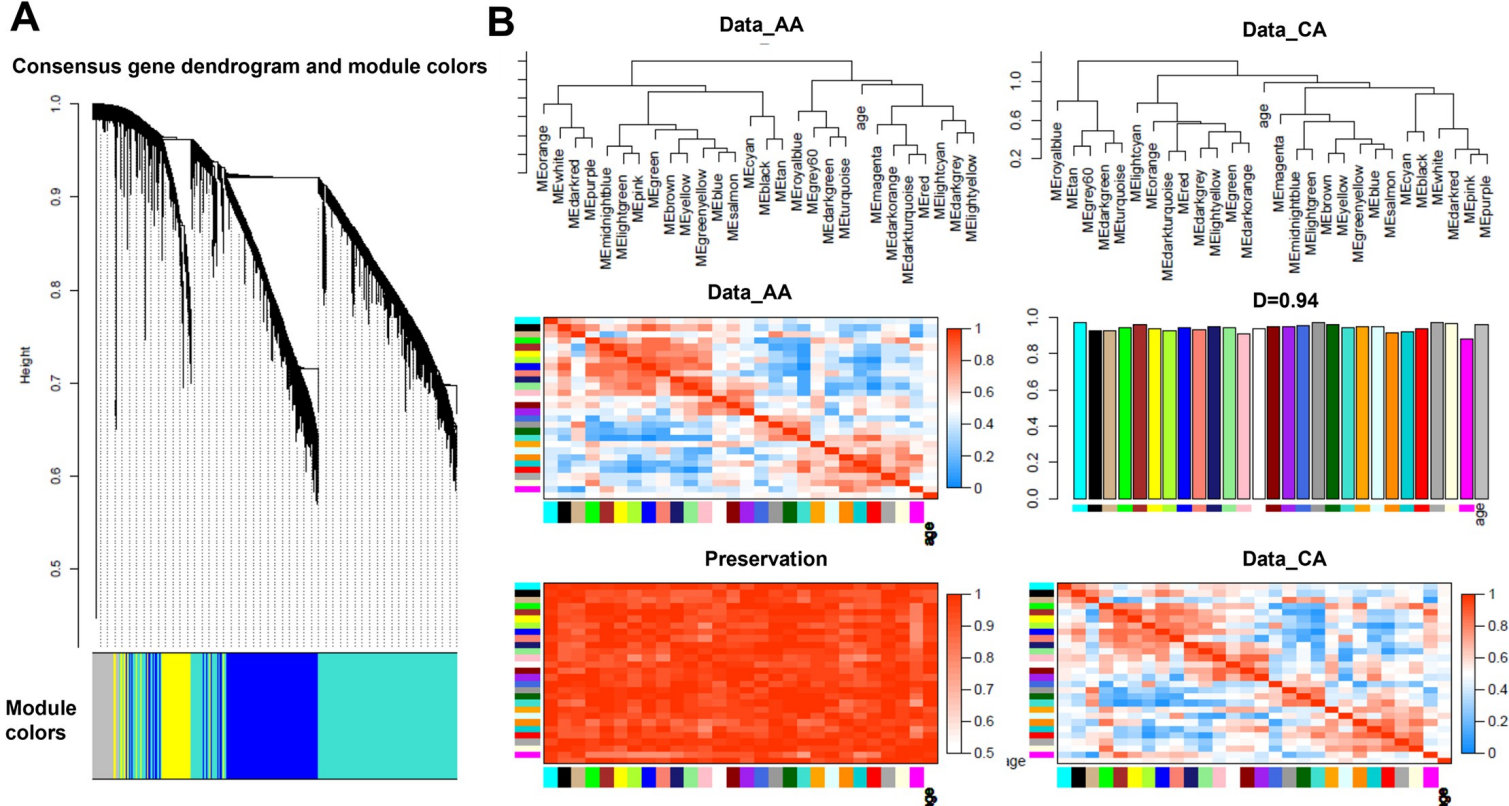
Consensus analysis of African Americans (AA) and Caucasians (CA) in the cohort of GSE 141910. (A): Consensus gene dendrogram and module colors using data from African Americans and Caucasians. The consensus gene dendrogram was obtained by average linkage using a pair-wise weighted correlation metric, and clustered according to a topological overlap metric into modules. Assigned modules were colored at the bottom. (B): Summary plot of consensus eigengene networks and their differential analysis from AA and CA groups. The top panels showed the clustering of consensus eigengenes in the two groups. The heat maps in the middle and bottom panel showed the correlation of eigengene network between the two groups, and the bar graph in the middle panel showed the mean preservation of adjacency for each eigengene to other eigengenes. The D value was calculated as the arithmetic mean of these measurements.

To evaluate the racial effects of the genetic change in this patient cohort, we separated the dataset into two groups: African Americans (AA) and Caucasian Americans (CA). As shown in Fig 5A, a gene dendrogram was obtained by average linkage of hierarchical clustering for AA and CA groups. Gene expression similarity was determined by pair-wise weighted correlation metric and clustered using a topological overlap metric. Gene modules are colored at the bottom. The comparison of eigengene and group module characteristics showed an overall similarity of 0.94 (Fig 5B).

**Fig 6:**
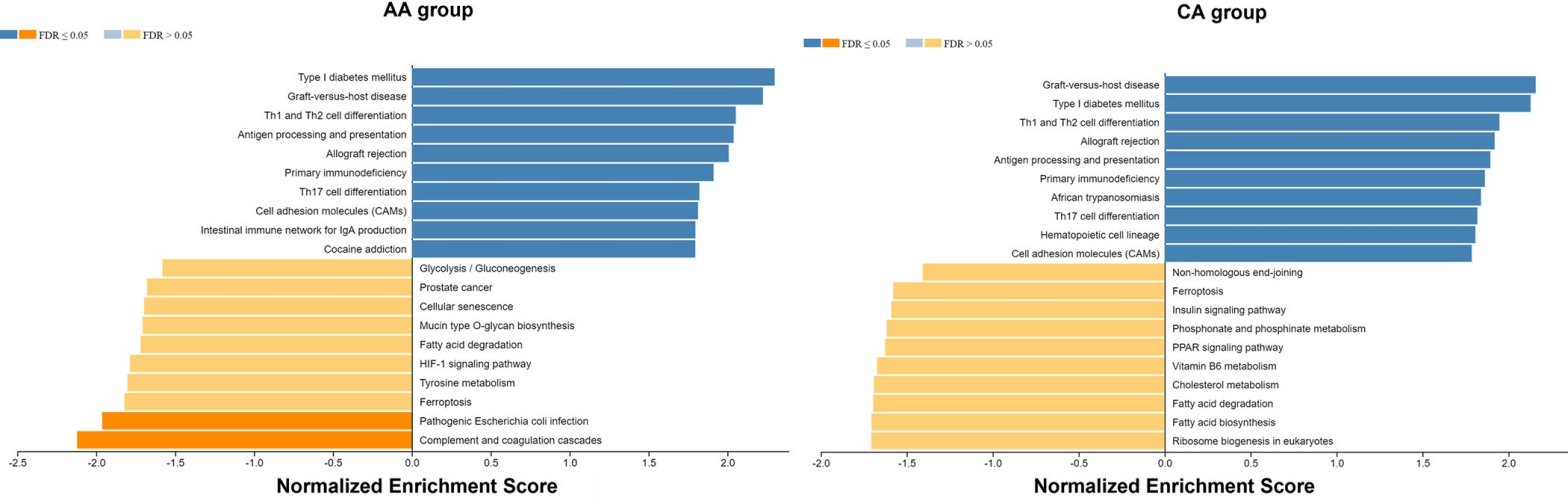
Gene expression and signaling pathway changes in African American (AA) and Caucasian (CA) in DCM patients versus donors. The log_2_FoldChange data from AA and CA groups were derived from DESeq2 R program and separately used for KEGG pathway analysis. The top 10 upregulated and downregulated pathways in each group were presented in the figure. FDR, false discovery rate. FDR<0.05 indicates significant change.

To further study signaling pathway changes in DCM patients from the AA and CA groups, we compared the overall gene expression change and performed a GSEA analysis using the Web-based Gene Set Analysis tool kit (www.webgestalt.org). The log_2_FoldChange in DCM patients compared to donor controls were derived from the counts data from each group using Deseq2 R-package as we previously described [44]. The log_2_FoldChange of genes in AA and CA groups were for KEGG pathway analysis. As shown in Fig 6, the top 10 upregulated signaling pathways in the AA and CA groups were highly overlapped, though the p values were modestly different. The 10 downregulated pathways in both groups were mostly not significant (FDR>0.05) except for the pathogenic E Coli infection and complement and coagulation cascades pathways in the AA group. These data suggested that there were no significant differences between the AA and CA groups in terms of gene expression and pathway changes during DCM.

### Na/K-ATPase reduction in patients with dilated cardiomyopathy

**Fig 7:**
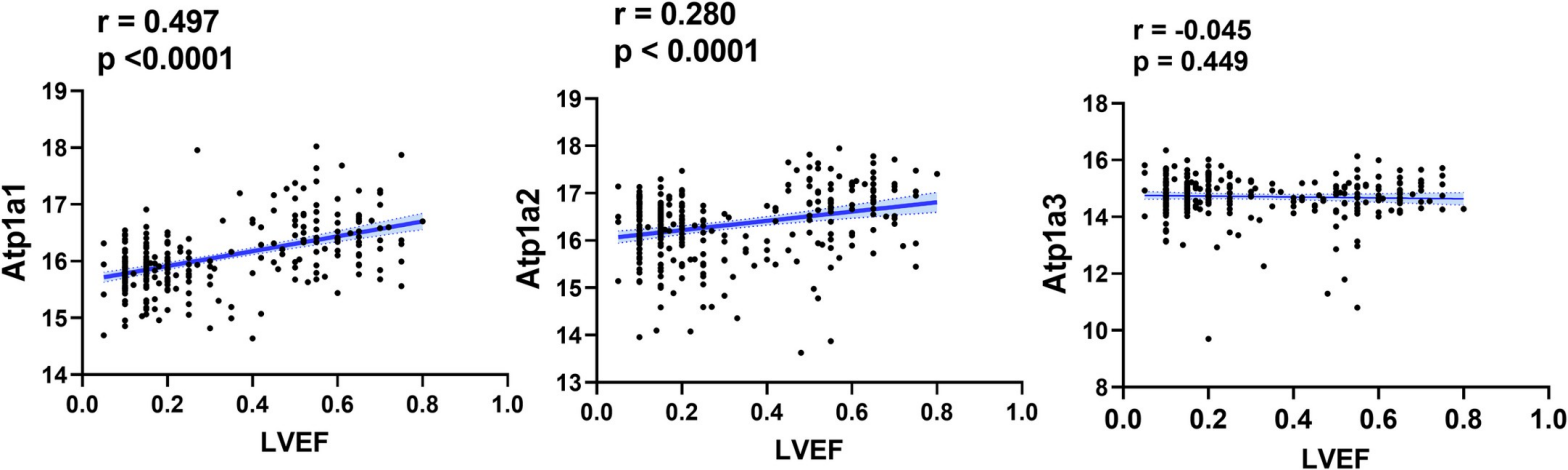
Relationship between Na/K-ATPase gene expression and heart function. The correlation between Na/K-ATPase α1, α2, and α3 gene expression and left ventricle ejection fraction (LVEF) was analyzed and presented in the figure. The scatter plot was created using GraphPad, and linear regression curves with 95% confidence (shadowed area) are shown in blue. Gene expression data of Na/K-ATPase (α1, α2, α3) genes and LVEF were obtained from published dataset (GSE141910).

Our previous studies in animals showed that Na/K-ATPase α1 reduction caused cardiac cell apoptosis and tissue fibrosis and was closely related to cardiac dysfunction [19, 20]. To evaluate the role of Na/K-ATPase in human cardiac dysfunction, we analyzed Na/K-ATPase gene expression and LVEF data in the above dataset of heart failure patients and their donors. As shown in Fig 7, Na/K-ATPase α1 expression was significantly correlated with LVEF in this cohort, while α2 had a relatively weaker correlation and α3 was not significantly correlated with LVEF. We then compared the Na/K-ATPase expression level in DCM patients (n = 166) to their non-failing donors (n = 166), the result showed that DCM patients had significantly lower expression of Na/K-ATPase (16.53±0.04 in donors and 15.82±0.04 in DCM patients, p<0.01). We also compared the Na/K-ATPase expression levels between African Americans and Caucasians and found no significant differences in both donor group and patient group.

**Fig 8:**
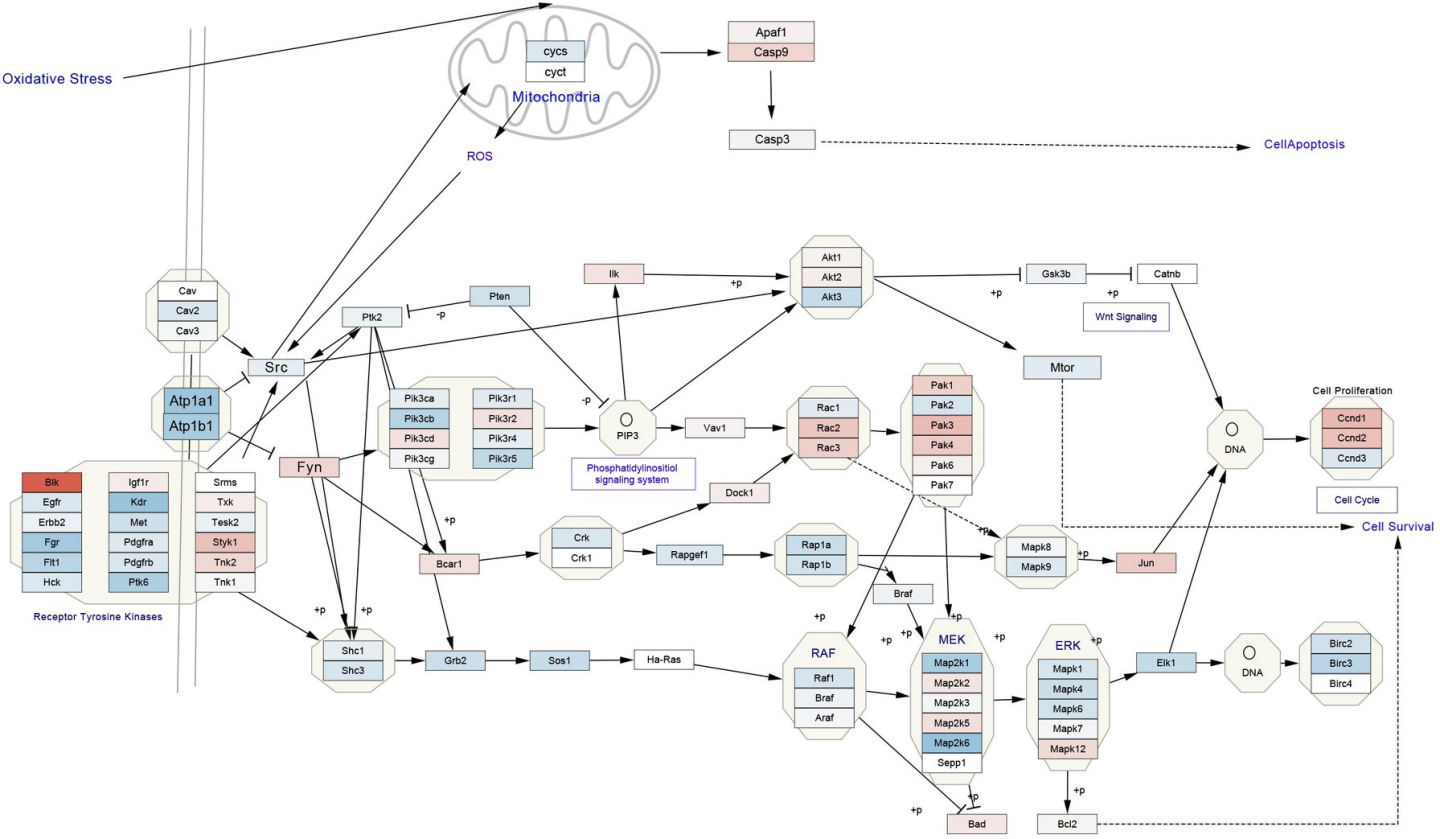
Mapping of gene expression change on Na/K-ATPase/Src signaling pathway. The log_2_FoldChange data from DCM patients versus donors and the corresponding gene names were mapped to the Na/K-ATPase/Src signaling pathway (Wikipathway WP5051) using Cytoscape. The log_2_FoldChange data were presented as continuous colors. The blue color indicates a downregulation and red color indicates an upregulation of the gene.

To understand how Na/K-ATPase was regulated in heart failure patients, we analyzed the potential regulators of genes in magenta module using ChEA3 transcriptional factor enrichment analysis as described in the Methods section. From this analysis, we found that three transcriptional factors (TFs) (BHLHE-40, RFX1, and EOMES) can directly regulate Na/K-ATPase α1 gene (ATP1A1) expression. To verify that these TFs indeed contribute to the regulation of Na/K-ATPase gene and other genes in this gene module, we compared the expression level of the three TFs in the heart-transplant DCM patients and their donors from the RNA-sequencing data and found that EOMES level was significantly increased to more than two-fold in DCM patients compared to that in non-heart failure donors, but BHLHE-40 and RFX1 were not significantly changed. To study how Na/K-ATPase-related signaling pathway was changed in DCM patients, we uploaded the log_2_FoldChange data derived from DESeq2 analysis to the Cytoscape program and searched against the Na/K-ATPase/Src wikipathway (WP5051). As shown in Fig 8, the blue color indicates decreased expression of the corresponding gene while red color indicates increased gene expression, which showed that some cell survival-related genes such as Egfr and other kinases were downregulated, while cell apoptosis-related genes such as Casp9 were upregulated.

## Discussion

WGCNA is a useful tool that can define gene modules, intramodular hubs, and network nodes and allow the study of relationships between co-expression modules. A gene module generally refers to a group of genes with similar expression patterns, and they tend to be functionally related and co-regulated [15, 45, 46]. Our current work used a large dataset of heart failure patients and identified several gene modules and signaling pathways that are closely related to DCM and reduced LVEF. The genes included in these modules are enriched in small molecule transport, fibrosis, and metabolic pathways. A significant finding is that there is no significant association between any gene modules and the ethnic groups. We performed gene module consensus analysis and compared the gene expression and pathway changes in African American and Caucasian patients and showed more similarity than disparity between the two groups of patients. The complexity of genetic factors that impact the persistence of health disparities between blacks and whites has been observed and has affected treatment recommendations, presenting a challenge of epic proportion for the medical community to tackle. On the other hand, African Americans or blacks in general have been underrepresented in many clinical trials for heart failure treatment, and the clinical outcome were less representative for blacks and consequently affect the usage of certain drugs in this population [47]. However, our data analysis results suggest that the development of reduced LVEF or DCM is associated with similar gene and pathway changes in African Americans and in Caucasians despite the differences in their genetic variations. This phenomenon should be considered in the strategy of DCM treatment in different ethnic groups, and there may be no reason to limit the use of the available drugs for African Americans even though the clinical data were not representative to this population. To this note, including more African Americans in future clinical trials is critical given that the heart failure prevalence in African Americans is significantly higher than Caucasians [9].

Among the genes that were changed in heart failure patients compared to non-failing donors, we specifically looked at the expression level of Na/K-ATPase, which is a membrane protein that plays an important role in energy metabolism and membrane potential in muscle cells. It has been previously reported that Na/K-ATPase concentration and activity were reduced in patients with heart failure, and cardiac ejection fraction was correlated with the amount of Na/K-ATPase [16, 21–23]. Since these studies have smaller patient numbers, we took the advantage of the published datasets with a large number of patients on GEO Profiles from NCBI and analyzed the data of Na/K-ATPase gene expression and its relationship with heart failure. It is noticed that the ~18% reduction in mRNA level of ATP1A1 was less than the protein amount change (~30%) reported in earlier studies [16, 23]. However, it makes sense considering the cumulative effect in the translational process. The Na/K-ATPase reduction was also observed in other datasets of DCM patients as well as in ischemic heart failure patients (Gene datasets GSE1145 and GSE26887, which can be found in GEO profiles website: https://www.ncbi.nlm.nih.gov/geoprofiles/5123633 and https://www.ncbi.nlm.nih.gov/geoprofiles/87379539). Our previous work has demonstrated that reduction of Na/K-ATPase causes tissue fibrosis and cell apoptosis in different cell types and mouse models of uremic cardiomyopathy [19, 20, 48]. Here, we showed that in DCM patients Na/K-ATPase reduction is associated with decreased LVEF, which is consistent with our previous studies on Na/K-ATPase reduction-induced cardiac dysfunction [19, 20].

Regulation of Na/K-ATPase involves transcriptional, translational, and post-translational mechanisms [49, 50]. We have previously reported that cardiotonic steroids such as ouabain can regulate Na/K-ATPase expression at the translational level [48]. The current study suggested that TFs may also be involved in Na/K-ATPase regulation in DCM patients. TFs are important regulators of gene expression. In human tissue, gene expression is controlled by about 1600 TFs [51]. A single TF can regulate multiple genes and a single gene could be simultaneously regulated by different TFs, and thus forms a regulatory network of TFs and their target genes. Since TFs are usually upstream regulators of gene expression, manipulation of the identified transcription factors could provide therapeutic targets for gene dysregulation in diseases. The ChEA3 TF enrichment analysis provides a platform to allow users to input their interested gene list and identify potential TFs that may coordinate the regulation of the listed genes [41]. The EOMES is a T-box transcriptional factor that is essential for embryonic cardiac development at mesoderm stage [52–54]. The increase of EOMES and decrease of Na/K-ATPase α1 in the DCMpatients may suggest a role of the transcription factor in regulation of DCM-related gene expressions.

## Limitations

The current study was based on one published database and the RNA-sequencing data may have batch effect. Since we used published dataset for this analysis, some information about the original experimental design such as inclusion and exclusion criteria as well as clinical presentations of these patients before heart transplant was not available. Future analysis using more datasets of DCM patients is needed to confirm current findings. In addition, we noticed that the donor group has a high hypertension rate (102 out of 166). Since hypertension could causes gene expression changes that are related with cardiac remodeling, the comparison of the donor and patients may not fully reflect the difference between the heart failure patients and normal healthy individuals. However, we think that our results still revealed important gene expression and signaling pathway changes between dysfunctional heart and functional heart.
